# PDE2A Is Indispensable for Mouse Liver Development and Hematopoiesis

**DOI:** 10.3390/ijms21082902

**Published:** 2020-04-21

**Authors:** Federica Barbagallo, Valentina Rotilio, Maria Rita Assenza, Salvatore Aguanno, Tiziana Orsini, Sabrina Putti, Andrea M. Isidori, Andrea Lenzi, Fabio Naro, Luciana De Angelis, Manuela Pellegrini

**Affiliations:** 1 Department of Experimental Medicine, Sapienza University, 00161 Rome, Italy; federica.barbagallo@uniroma1.it (F.B.); andrea.isidori@uniroma1.it (A.M.I.); andrea.lenzi@uniroma1.it (A.L.); 2 Anatomical, Histological, Forensic and Orthopedic Sciences, Sapienza University, 00161 Rome, Italy; valentina.rotilio@uniroma1.it (V.R.); mariarita.assenza@cnr.it (M.R.A.); salvatore.aguanno@uniroma1.it (S.A.); fabio.naro@uniroma1.it (F.N.); luciana.deangelis@uniroma1.it (L.D.A.); 3 Institute of Biochemistry and Cell Biology, National Research Council, 00015 Monterotondo, Rome, Italy; tiziana.orsini@cnr.it (T.O.); sabrina.putti@cnr.it (S.P.)

**Keywords:** phosphodiesterase 2A, fetal liver, cAMP, ICER, apoptosis, hematopoiesis

## Abstract

Phosphodiesterase 2A (PDE2A) is a cAMP-cGMP hydrolyzing enzyme essential for mouse development and the *PDE2A* knockout model (*PDE2A*^−/−^) is embryonic lethal. Notably, livers of *PDE2A*^−/−^ embryos at embryonic day 14.5 (E14.5) have extremely reduced size. Morphological, cellular and molecular analyses revealed loss of integrity in the *PDE2A*^−/−^ liver niche that compromises the hematopoietic function and maturation. Hematopoietic cells isolated from *PDE2A*^−/−^ livers are instead able to differentiate in in vitro assays, suggesting the absence of blood cell-autonomous defects. Apoptosis was revealed in hepatoblasts and at the endothelial and stromal compartments in livers of *PDE2A*^−/−^ embryos. The increase of the intracellular cAMP level and of the inducible cAMP early repressor (ICER) in liver of *PDE2A*^−/−^ embryos might explain the impairment of liver development by downregulating the expression of the anti-apoptotic gene Bcl2. In summary, we propose *PDE2A* as an essential gene for integrity maintenance of liver niche and the accomplishment of hematopoiesis.

## 1. Introduction

During most of prenatal development, the fetal liver (FL) provides the specific niche required for hematopoiesis. Its components, hepatoblasts, resident macrophages, fibroblasts, vascular smooth muscle, stromal and endothelial cells participate with extracellular matrix (ECM) and humoral factors to its generation [1]. In the mouse, at embryonic day 11.5 (E11.5), hematopoietic stem cells (HSC), after their formation in the yolk sac, aorta-gonad-mesonephros region, and placenta, migrate in FL attracted by chemical signals. In FL between E12 and E16, HSC dramatically increase in number and differentiate into mature hematopoietic cells, which are necessary to sustain the ongoing growth of the embryo [2]. Around E16, the hematopoietic function of FL starts to decline in favor of the bone marrow and the liver begins to acquire specific metabolic functions, which include carbohydrate and lipid metabolism [3].

A plethora of genetic alterations affecting liver development has been reported to compromise hematopoiesis. By examining in vitro proliferation and differentiation of the hematopoietic committed progenitors it is possible to distinguish if defects are being housed in the niche or in the hematopoietic precursors themselves [4,5,6]. Phosphodiesterases (PDEs) are essential components of cellular signaling that regulate the response to several stimuli (hormones, neurotransmitters) by modulating intracellular levels of cyclic nucleotides. There are eleven mammalian PDEs families encoded by numerous genes and each of them can give rise to several splicing variants [7].

PDEs are expressed in different combinations in tissues and in cellular compartments allowing the proper regulation of cyclic nucleotides signaling. Some PDEs are cAMP or cGMP-specific or, as in the case of phosphodiesterase 2A (PDE2A), they can hydrolyze both cAMP and cGMP. In the adult mouse, PDE2A has a widespread tissue distribution but brain, heart, liver and adrenal gland show the greatest level of expression. Mouse development is compromised in *PDE2A* knockout embryos [8] and we recently also documented cardiac defects in this mouse model [9]. PDE2A deficiency in fetal heart causes the increase of cAMP level and the expression of a cAMP-dependent transcriptional repressor, which in turn reduces the expression of genes essential for cardiomyocyte differentiation [9]. The role of PDE2A in liver development and hematopoiesis has been not yet established, but in the adult the role of cyclic nucleotides in regulating protein kinase activity and gene transcription for key liver functions is well documented [10,11].

Here we report a new role of PDE2A in FL. The absence of this enzyme severely affects the development of this organ at the structural, cellular and molecular levels with severe consequences on its role in prenatal hematopoiesis. We show for the first time the gross aberrations in *PDE2A*^−/−^ fetal liver and hematopoiesis, indicating that PDE2A is primarily required for liver development and secondarily for hematopoietic development.

## 2. Results

### 2.1. PDE2A Deficient Embryos Display Fetal Liver Defects

*PDE2A*^+/−^ mice are normal and fertile, but do not produce viable homozygous mutants (*PDE2A*^−/−^), indicating that PDE2A deficiency is lethal [8,9].

As we previously reported, the mutant mice die in utero at E15.5. At E14.5, *PDE2A*^−/−^ embryos are small, pale and display cardiac septum defects [9]. Many embryos present diffuse hemorrhages (40% out of 86 total *PDE2A*^−/−^ embryos) and edema in the head region (30% out of 86 total *PDE2A*^−/−^ embryos).

**Figure 1:**
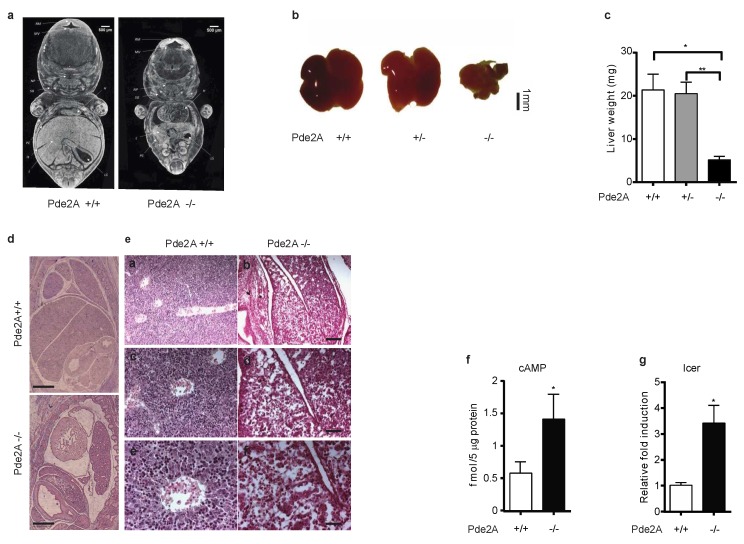
Morphological defects in liver of *PDE2A*^−/−^ embryos. (**a**) Volume rendering visualization of coronal sections obtained from E14.5 *PDE2A*^+/+^ and *PDE2A*^−/−^ embryos. Sections were chosen referring to the stomach lumen (*LS*) volume. *H* heart, *L* liver, *PC* peritoneal cavity, IS interlobular space, *MV* mesencephalic vesicle, *SG* submandibular gland, *RM* roof of midbrain, NP nasopharynx. (**b**) Representative stereomicroscope picture of livers isolated from E14.5 *PDE2A*^+/+^, *PDE2A*^+/−^ and *PDE2A*^−/−^ embryos. The liver of *PDE2A*^−/−^ is hypoplastic. (**c**) Liver weight compared at E14.5 in *PDE2A*^+/+^, *PDE2A*^+/−^ and *PDE2A*^−/−^ embryos. (**d**) Histological analysis of E14.5 embryos stained with H&E. Scale bar 500 µm. (**e**) Histological analysis of E14.5 liver sections stained with H&E. Scale bars: 200 µm (**a**,**b**), 100 µm (**c**,**d**) and 50 µm (**e**,**f**). (**f**) cAMP level in E 14.5 *PDE2A*^+/+^ and *PDE2A*^−/−^ livers. (**g**) Expression of ICER in livers of E14.5 *PDE2A*^+/+^ and *PDE2A*^−/−^ mice evaluated by qRT-PCR. At least *N* = 3 embryos/genotype were analyzed in each experiment. * *P* < 0.05; ** *P* < 0.01.

To further characterize the gross phenotype, embryos were analyzed by computational tomography (micro-CT) (Figure 1a and Supplemental Videos).

Micro-CT scanning confirmed the cardiac defects at E14.5 previously described [9] and revealed new abnormalities affecting organs morphology and positioning (Figure 1a and Supplemental Videos). The clearest morphological abnormality was the smallest dimension of *PDE2A*^−/−^ liver (Figure 1a,b), confirmed by weight, which in *PDE2A*^−/−^ is 25% of that of *PDE2A*^+/−^ and *PDE2A*^+/+^ littermates (Figure 1c) and by volumetric (µm^3^) comparison of the ratio between liver and body volumes (5:1) (*PDE2A*^+/+^ 0.1110 ± 0.004933 and *PDE2A*^−/−^ 0.02169 ± 0.001459, *P* < 0.0001, *N* = 3). Furthermore, no evident lobes division was observed in *PDE2A*^−/−^ embryos liver (Figure 1b and Supplemental Video). Lungs and kidneys of *PDE2A*^−/−^ embryos appeared in lowered position relatively to the longitudinal axis (Figure 1a and Supplemental Video). Compared to wild type mice, at E11.5 the development of knockout liver is normal, but differences appear at E12.5 (Supplemental Figure S1a). With time, the differences increase and become clear in E14.5 embryos (Figure 1 and Supplemental Figure S1a).

Histological examination of the livers of *PDE2A*^−/−^ E14.5 embryos displays loss of the normal liver structure as the central vein is not recognizable in the lobule and the cells are less densely packed with increased empty space among them. Erythroblasts, easily detected at E14.5 in normal livers thanks to their hyperchromatic nuclei, are poorly represented in *PDE2A*^−/−^ embryos (Figure 1d,e).

The absence of PDE2A in the cells could impact on cellular cAMP concentration if not compensated by the activity of other PDEs. To verify whether *PDE2A*^−/−^ embryos had an abnormal cAMP accumulation, we measured the level of cAMP in the homogenate of livers of *PDE2A*^+/+^ and *PDE2A*^−/−^. Figure 1f shows that the level of cAMP in liver of E14.5 *PDE2A*^−/−^ embryos is significantly higher compared to the levels detected in the liver of *PDE2A*^+/+^ embryos. Moreover, in liver of E14.5 *PDE2A*^−/−^ embryos the mRNA level of ICER, a cAMP-dependent transcription repressor, was up-regulated compared to heterozygous and wild type mice (Figure 1g), as previously observed in PDE2A deficient embryonic hearts [9].

Altogether, these results suggest that PDE2A is a master gene that orchestrates liver development during embryogenesis, possibly controlling cAMP and ICER concentration.

### 2.2. Hepatic Markers Are Reduced Whereas Stromal and Endothelial Markers Are Increased in Liver Of PDE2A Mutant

We first investigated the impact of PDE2A absence by comparing mutant, wild type and heterozygous developing livers for the level of markers relevant to hepatocytes differentiation and function [3].

**Figure 2:**
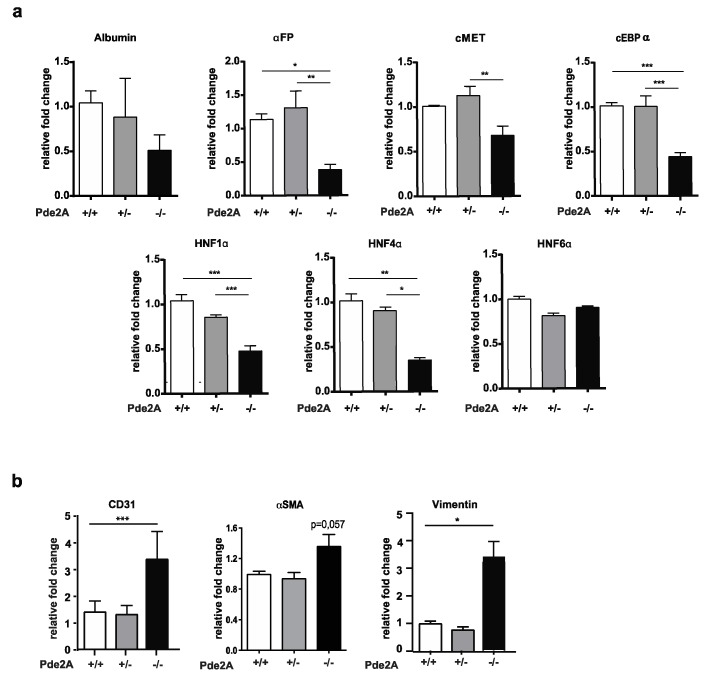
Transcript modulation in liver maturation of *PDE2A*^−/−^ embryos. qRT-PCR in E14.5 liver embryos shows reduction of expression of liver differentiation markers and increase of stromal and endothelial markers relative to GAPDH expression. (**a**) Relative mRNA level of albumin, α-fetoprotein and transcription factors cMET, cEBPα, HNF1, HNF4 and HNF6 in liver of *PDE2A*^−/−^ embryos. (**b**) Relative mRNA level of CD31, vimentin and α-SMA in liver of *PDE2A*^−/−^ embryos. At least *N* = 3 embryos/genotype were analyzed in each experiment. * *P* < 0.05; ** *P* < 0.01; *** *P* < 0.001.

Figure 2a shows that livers of *PDE2A*^−/−^ embryos have reduced levels of albumin and α-fetoprotein (α-FP) respect to livers of *PDE2A*^+/−^ and *PDE2A*^+/+^ embryos. The same outcome results examining the expression of hepatocyte growth factor receptor (cMet), which reduction reaches 20% and of CCAAT/enhancer binding protein alpha (cEBPα) and some hepatocyte nuclear factors (HNF1α, and HNF4α) that are halved. The expression of these factors in *PDE2A*^−/−^ livers starts to decline at E12.5 (Supplemental Figure S1b) and becomes statistically significant at E14.5.

To investigate whether PDE2A activity directly affects hepatic marker expression, isolated hepatic cells from E14.5 C57BL/6 embryos were treated for 48 h with 10 µM of the selective PDE2A inhibitor erythro-9-(2-hydroxy-3-nonyl)adenine (EHNA). As shown in Supplemental Figure S2 no major differences were observed in gene expression analysis after PDE2A inhibition, indicating that PDE2A activity is dispensable for hepatoblast differentiation, at least in vitro.

Afterwards, the impact of PDE2A was evaluated on endothelial and stromal cells which contribute to hematopoietic development in concert with hepatic cells. Figure 2b shows a significant increase of CD31 endothelial marker and of the stromal markers α-smooth muscle actin (α-SMA) and vimentin in *PDE2A *^−/−^ compared to *PDE2A*^+/−^ and *PDE2A*^+/+^ livers.

Overall, these results suggest that in vivo PDE2A contributes to hepatoblast differentiation/survival and inhibits stromal and endothelial cells differentiation.

### 2.3. Increased Apoptosis in PDE2A^−/−^ Fetal Livers

The small dimension of the livers of *PDE2A* knockout embryos and the histological data indicate a reduced cellularity of the organ. In the livers of knockout embryos, the number of cells is 25 times lower compared to heterozygote or wild type animals. This implies an increased rate of cell death and/or a decreased rate of cell proliferation.

To investigate these two possibilities, we evaluated cells dissociated from livers of E14.5 wild type, heterozygous and mutant mice by flow cytometry for their phase in the cell cycle.

**Figure 3:**
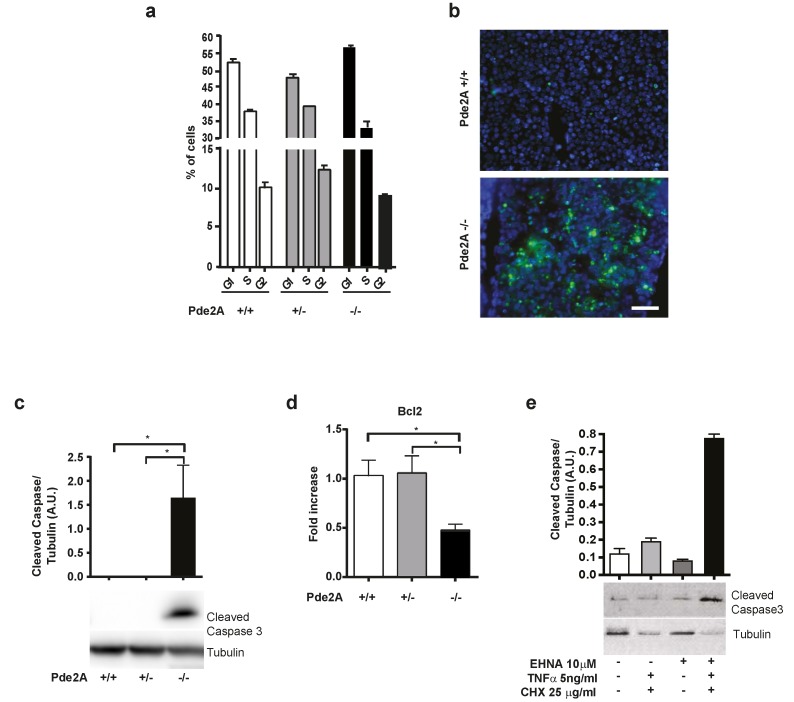
Apoptosis is increased in liver of *PDE2A*^−/−^ embryos. (**a**) Cells dissociated from liver of E14.5 *PDE2A*^+/+^, *PDE2A*^+/−^ and *PDE2A*^−/−^ embryos display cell cycle similarities by flow cytometry analysis. (**b**) TUNEL staining shows increased apoptosis in livers sections derived from E14.5 *PDE2A*^−/−^ embryos respect to *PDE2A*^+/+^ embryos. Scale bar: 50 µm. *N* = 3 embryos/genotype. (**c**) Representative western blot analysis of cleaved caspase-3 expression in liver extracts of E14.5 *PDE2A*^+/+^, *PDE2A*^+/−^ and *PDE2A*^−/−^ embryos. Densitometry is shown relative to tubulin expression. *N* = 3 embryos/genotype. (**d**) qRT-PCR in E14.5 liver embryos showing Bcl2 expression. *N* = 3 embryos/genotype. (**e**) E14.5 liver cells isolated from C57BL/6 embryos enter apoptosis after TNFα (5 ng/mL) and CHX (25 µg/mL) treatments if pretreated with the PDE2A inhibitor EHNA (10 µM). Apoptosis was evaluated by cleaved caspase-3 in western blots. Densitometry analysis relative to tubulin is shown. *N* = 2 embryos. * *P* < 0.05.

The liver of *PDE2A*^−/−^ embryos had the same proportion of cells in G1, S and G2 phase (Figure 3a). Moreover, the proportion of proliferating cells was similar as evaluated by Ki67 staining in histological sections (not shown).

On the contrary, TUNEL assay in sections of *PDE2A*^−/−^ livers at E14.5 displayed a clear increment of apoptotic cells compared to *PDE2A*^+/+^ livers (Figure 3b). Apoptosis affected the liver while was not appreciable in other organs except for the cardiac septum [9]. In keeping with this result, western blots analysis of lysates from mutant livers at E14.5, but not from livers of wild type and heterozygous embryos, displayed a strong positive band of cleaved caspase-3 (Figure 3c), a marker of apoptosis. Furthermore, in the liver of mutant embryos the level of transcript of the anti-apoptotic factor Bcl2 was lower respect to wild type and heterozygous embryos (Figure 3d).

To investigate whether PDE2A activity prevents apoptosis of liver cells, we used EHNA in cultures of fetal liver cells isolated from C57BL/6 embryos. Treatment for 48 h with EHNA (10 µM) did not increase apoptosis. Nevertheless, EHNA (10 µM) treated cells underwent apoptosis and displayed caspase-3 activity if the inhibitor was administered together with tumor necrosis factor-alpha (TNFα, 5 ng/mL) and low doses of cycloheximide (CHX, 25 µg/mL) (Figure 3e) for the last 24 h, suggesting that PDE2A inhibition increases the sensitivity of liver cells to apoptotic stimuli.

Taken together, these data indicate that liver hypoplasia in *PDE2A*^−/−^ embryos is caused by apoptotic increase rather than decrease of cellular proliferation and that PDE2A by reducing the intracellular concentration of cAMP/ICER, may play a role in protecting liver cells from apoptotic factors during development.

### 2.4. Hepatoblasts, Endothelial and Stromal Cells Undergo Apotosis in Livers of PDE2A^−/−^ Embryos^−^

**Figure 4:**
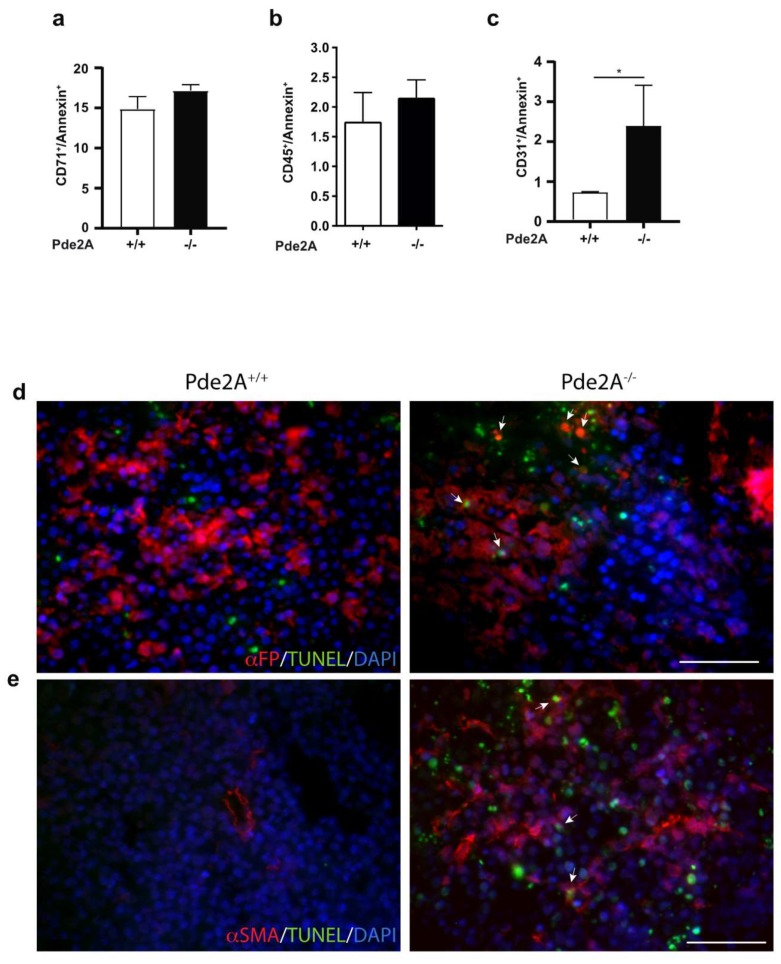
The niche is compromised in liver of *PDE2A*^−/−^ embryos. (**a**–**c**) Flow cytometry analysis of liver cells isolated from E14.5 *PDE2A*^+/+^ and *PDE2A*^−/−^ embryos, stained with Annexin-V and antibodies against CD71 (**a**), CD45 (**b**), CD31 (**c**) markers. Histogram showing the percentage of double stained cells reveals an increase of endothelial cell apoptosis in *PDE2A*^−/−^ embryos. *N* = 3 embryos/genotype. * *P* < 0.05. (**d**,**e**) Immunofluorescence of E14.5 liver sections stained with α-FP and α-SMA antibodies (red) and with TUNEL assay. Nuclei were counterstained with DAPI (blue). Arrows indicate double stained cells. Scale bar 50 µm. *N* = 3 embryos/genotype.

To investigate which cell types undergo apoptosis in livers of *PDE2A*^−/−^ embryos, flow cytometry and histological analyses were performed in E14.5 mutant and wild type embryos. Isolated CD71^+^ immature red blood cells and CD45^+^ leucocytes [12,13] co-stained with the apoptotic cell marker Annexin-V, displayed a similar pattern in wild type and mutant embryos (Figure 4a,b). On the contrary, apoptosis was significantly higher in endothelial cells (CD31^+^/Annexin-V^+^) obtained from the liver of *PDE2A*^−/−^ embryos (Figure 4c). Apoptosis of hepatoblasts and stromal cells was evaluated in paraffin liver sections. In *PDE2A*^−/−^ but not in *PDE2A*^+/+^ livers, a portion of hepatoblasts (α-FP positive, Figure 4d) and stromal cells (α-SMA positive, Figure 4e) was stained with TUNEL assay indicating that in mutant animals, apoptosis reduces the number of these cells. Furthermore, the staining with α-SMA revealed that in mutant livers positive cells are more numerous and disseminate.

These results strongly indicate that in *PDE2A*^−/−^ embryos, the absence of PDE2A induces apoptosis in cells of different lineages that overall forms the liver niche.

### 2.5. Impairment of Hematopoietic Differentation in PDE2A^−/−^ Embryos

**Figure 5:**
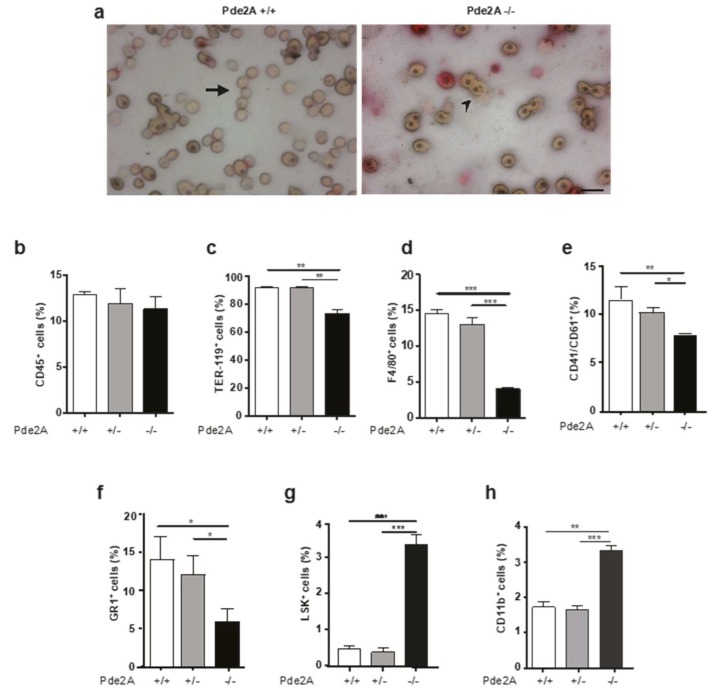
Impairment of blood cells maturation in *PDE2A*^−/−^ embryos. (**a**) Smear of blood from E14.5 *PDE2A*^+/+^ and *PDE2A*^−/−^ embryos. Arrowhead indicates nucleated red blood cells originated in the yolk sac; arrow indicates mature non-nucleated cells derived from liver. Scale bars: 20 µm. In *PDE2A*^−/−^ embryos the immature nucleated blood cells are predominant compared to *PDE2A*^+/+^. (**b**–**h**) Flow cytometry analysis of liver cells isolated from E14.5 *PDE2A*^+/+^, *PDE2A*^+/−^, *PDE2A*^−/−^ embryos, stained with antibodies against CD45, Ter119, F4/80, CD41/CD61, GR1 and LSK markers. Ter119, F4/80, CD41/CD61, GR1, that label mature erythrocytes, macrophages, megakaryoblasts and granulocytes respectively, show statistically significant reduction, whereas LSK and CD11b, detecting stem/progenitor cells, show a significant increase in *PDE2A*^−/−^ samples compared to *PDE2A*^+/+^ and *PDE2A*^+/−^ embryos. *N* = 4 embryos/genotype. * *P* < 0.05; ** *P* < 0.01; *** *P* < 0.001.

In mouse embryos between E11.5 and E16.5, the liver is the primary hematopoietic organ [14,15]. Blood smears obtained from E14.5 normal peripheral blood display a great number of mature non-nucleated erythroid cells, which are mainly liver derived. In blood smears obtained from *PDE2A*^−/−^ embryos, erythroid cells are still predominantly nucleated and hence immature. Most likely, they are remnants of yolk sac origin suggesting an impairment of erythropoiesis in *PDE2A*^−/−^ embryos (Figure 5a). Furthermore, the number of blood cells collected from the umbilical cord of *PDE2A*^−/−^ embryos revealed a 4.5-fold reduction compared to *PDE2A*^+/+^.

The previous results prompted us to investigate the hematopoietic development analyzing with flow cytometry cells isolated from the liver of E14.5 wild type, heterozygous and mutant embryos stained with antibodies directed to specific hematopoietic lineages (Figure 5b–h).

The relative proportion of CD45 positive cells resulted similar to wild type in the liver of *PDE2A*^−/−^ embryos (Figure 5b). Instead, the staining for Ter119, a cell surface erythroid lineage marker expressed in terminally differentiating erythroblasts [16,17] showed the fall of erythroblasts percentage from 90% in the liver of *PDE2A*^+/+^ to 75% in the liver of *PDE2A*^−/−^ embryos (Figure 5c). Staining with F4/80, CD41/CD61 and Gr1, that label mature macrophages, megakaryoblasts and granulocytes respectively [13,18,19], showed a reduction of these cells in *PDE2A*^−/−^ livers (Figure 5d–f). No major differences were observed in lymphoid lineage labeled with B220 and CD3 markers (Supplemental Figure S3). These results suggest that in fetal livers of *PDE2A*^−/−^ embryos the number of mature blood cells of myeloid and erythroid lineages is lower.

On the other side, the percentage of the LSK population of stem/progenitor cells, identified by Lin^−^, Sca1^+^, c-Kit^+^ in fetal livers at E14.5 [20], increased in mutant embryos (Figure 5g), even if the absolute number of LSK was reduced proportionally to *PDE2A*^−/−^ liver size (*PDE2A*^+/+^ 74473 ± 3476 vs *PDE2A*^−/−^ 17548 ± 1168, *P* < 0.01). In agreement with this result, it was observed an increase in the percentage of CD11b positive cells (Figure 5h) that are also part of the progenitor population in fetal liver [12,21]. These results suggest that hematopoietic stem cells colonize and survive in *PDE2A*^−/−^ fetal liver, but apoptosis of the cells in their supporting niche compromises their differentiation.

**Figure 6:**
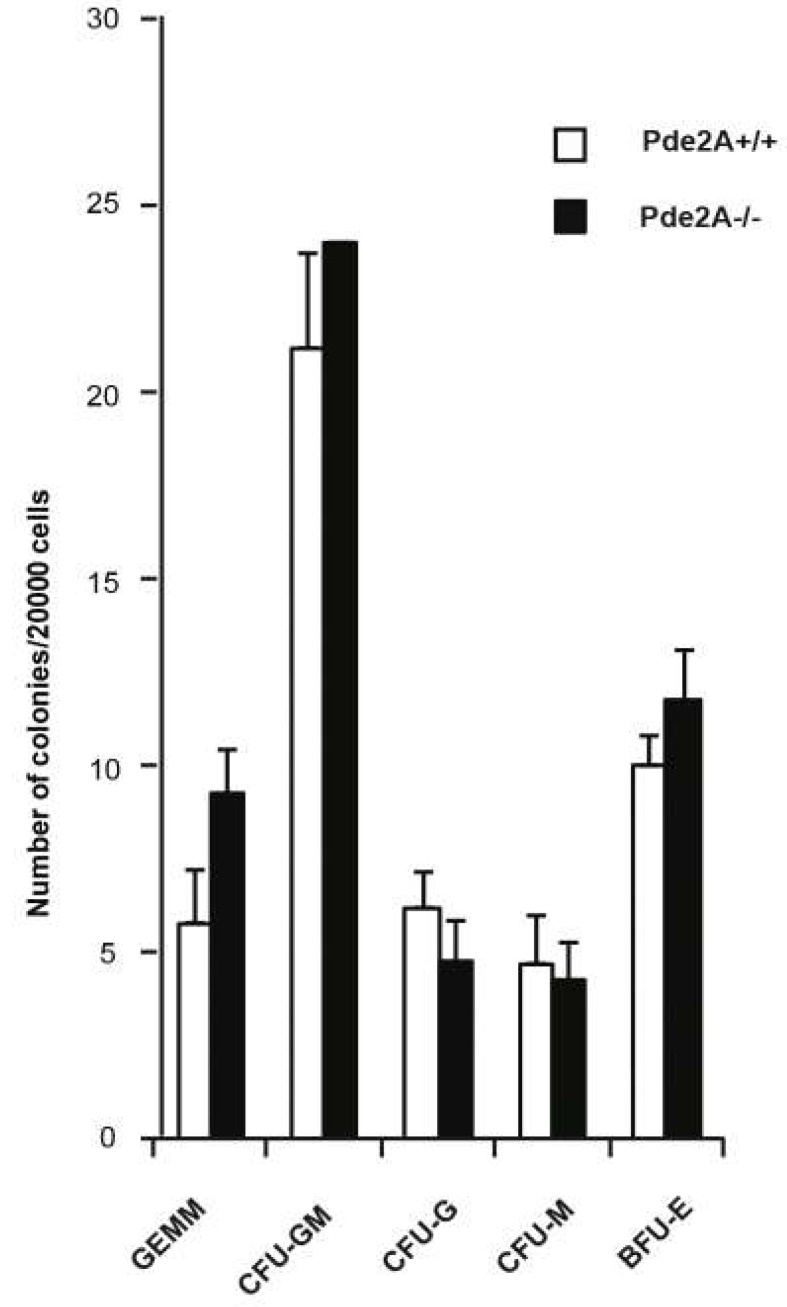
The maturation potential of hematopoietic progenitor/stem cells is preserved in *PDE2A*^−/−^ embryos. Colony-Forming-Cell (CFC) assay identifies an equivalent number of colonies in cells isolated from E14.5 *PDE2A*^+/+^ and *PDE2A*^−/−^ livers. The different types of colonies are labeled as follows: GEMM colony forming unit granulocyte-erythrocyte-megakaryocyte-monocyte; CFU-GM granulocyte-monocyte; CFU-G granulocyte; CFU-M monocyte; BFU-E Burst forming unit erythroid. At least *N* = 3 embryos/genotype were analyzed in each experiment in triplicates.

To further confirm this hypothesis, we compared the capacity of PDE2A^−/−^ and PDE2A^+/+^ hematopoietic progenitor cells of livers from E14.5 to develop into the erythroid and myeloid lineages in an in vitro methylcellulose colony assay [22]. The number of erythroid and myeloid colonies obtained from the two genotypes after 7 days of culture was equivalent (Figure 6), indicating that *PDE2A*^−/−^ hematopoietic stem cells are able to proliferate and differentiate when tested in vitro under proper conditions and even though originating from severely hypoplastic and immature livers, give rise to all canonical hematopoietic lineages.

## 3. Discussion

In this study, we show that the lack of PDE2A results in profound defects in early liver development. At the time of death, livers are hypocellular because of apoptosis and pale because the differentiation of mature blood cells from their progenitors is defective.

In parallel, we observed an increase of the stem/progenitor population, probably due to the lack of proper differentiation conditions. Nevertheless, the hematopoietic progenitors isolated from the livers of knockout embryos proliferate and differentiate normally in vitro giving rise to respective hematopoietic lineages. The increase in colony formation of *PDE2A*^−/−^ embryos did not correspond to the increase observed in their stem/progenitor cells, probably because the latest lost their full competence in vivo since they were hosted in a compromised environment at the peri-lethal stage of development.

PDE2A deficient embryos share similarities with other mouse models in which gene disruption causes abnormal liver development. The most similar picture is offered by *XBP-1* knockout mice [6]. XBP1 is a major component of the unfolded protein response (UPR) of the endoplasmic reticulum. Knockout mouse embryos die at mid-gestation because the hematopoietic progenitors do not generate mature blood cells for the inadequate environment in the hypoplastic liver, but they differentiate normally in vitro. However, XBP-1 is a cAMP-dependent factor and indeed we observed that *XBP-1* mRNA is upregulated in *PDE2A* mutants (data not shown) implying that this factor is unlikely responsible for the phenotype of *PDE2A*^−/−^ embryos. However, we cannot exclude that cAMP level in *PDE2A*^−/−^ embryos affects the activity of other UPR components.

*PDE2A* and *XBP-1* knockout embryos share a decreased expression of αFP, an early marker, which promotes the growth of liver cells and may contribute to the reduced liver size in both models.

Furthermore, *PDE2A*^−/−^ embryos have reduced expression of several liver specific transcription factors, essential to establish liver function (such as cEBPα, cMet, HNF1α, HNF4α) and of albumin.

All of these genes are essential for liver development. Albumin expression starts at E13.5 and later increases to fulfill its function as serum protein [23]. The expression of αFP starts at E11.5 in the liver and increases until birth, when is silenced [24,25]. cMet codifies for the receptor of hepatocyte growth factor (HGFR), which is essential for the survival of hepatocytes during development [26]. cEBPα is a factor involved in the commitment of the hepatoblasts to the hepatocytic and cholangiocytic lineages [27,28] and HNFs regulate the expression of factors relevant for the function of the fetal liver [29,30].

None of these genes appears to contain CREB binding sites in the promoter; however, we cannot exclude that cAMP pathways affect indirectly their expression. Since there are several critical pathways which control hepatogenesis such as TGFβ, Wnt, FGF, BMP signaling [31,32,33], it is possible that the increase of cAMP in PDE2A mutant mice disturbs dosage and/or signaling of cytokines and growth factors severely compromising liver development.

In vitro experiments of PDE2A activity inhibition in wild type fetal liver cells did not replicate the downregulation of specific hepatic transcripts observed in mutant liver in vivo. These results may be interpreted in different ways. It is possible that the timing of mRNA analysis was not correct or that the reduction of expression in vivo reflects the loss of hepatic cells which does not occurs in vitro. In alternative, the lack of downregulation of gene expression in vitro could be due to the inadequate cellular cross talk in this environment.

Interestingly, in the livers of mutant animals we found an increase of expression of endothelial and stromal markers. However, stromal cells more undergo to apoptosis and are displaced in the tissue, probably because apoptosis reduces the hepatic parenchyma, as previously reported [34].

Mice deficient for genes regulating the NF-kB pathway offer another example of phenotype with points in common with that of PDE2A mutants. During mid-fetal gestation, the embryos exhibit massive liver degeneration [5,35,36,37,38] indicating that NF-kB protects embryonic liver from apoptosis induced by TNFα, which is produced by hematopoietic cells [38,39,40,41,42]. We did not investigate the NF-kB pathway in *PDE2A*^−/−^ livers; however, hepatocytes from wild type livers undergo apoptosis when cultured with the PDE2A inhibitor EHNA and TNFα.

The absence of PDE2A is not compensated by the activity of other PDE members and this accounts for the high level of cAMP observed in liver of mutant embryos underlying the importance of this enzyme in balancing the cAMP signaling.

Agents that elevate cAMP stimulate apoptosis by activating PKA [42] in a variety of other systems, including thymocytes [41], immortalized primary granulosa cells [43,44], human mammary carcinoma cells [45] and various normal and transformed T and B cells [42]. In other cases, PKA activated by cAMP blocks apoptosis. Examples include aged neutrophils [46], macrophage cell lines exposed to exogenous nitric oxide [47], ovarian follicles [48] and T cells [49].

In *PDE2A*^−/−^ embryos of E14.5, we found that apoptosis hits specific cell populations such as hepatoblasts, endothelial and stromal cells, that constitute the niche supporting the efficient blood cell differentiation at this stage of development. It is plausible that the moderate apoptosis of these cells we see in the liver at this time is just the tail end of events occurred earlier.

Moreover, we found that the number of F4/80 macrophages is severely reduced in the fetal livers of *PDE2A*^−/−^ embryos. Erythroid cell differentiation in the erythroblastic island depends on macrophages in the fetal liver [18,50]. Thus, defects of macrophages may contribute to the impairment of red blood cell maturation in *PDE2A*^−/−^ embryos.

A high level of cAMP could promote apoptosis not only by activating PKA, but also by directly deregulating the transcription of cAMP-responsive genes such as ICER that represses the transcription of several CRE (cAMP responsive regulatory element) containing genes [51]. We recently reported the upregulation of the cAMP/ICER system in the hearts of *PDE2A*^−/−^ embryos or after PDE2A inhibition in vivo, in which like in the liver we observed increased apoptosis and modulation of several genes necessary for cardiogenesis [9]. Several examples indicate that ICER can induce apoptosis by downregulating the Bcl2 anti-apoptotic gene [52,53]. In *PDE2A*^−/−^ livers, ICER is upregulated and Bcl2 is downregulated, suggesting a mechanism through which liver cells undergo apoptosis.

In conclusion, in the mouse, the lack of PDE2A through the increase of cAMP could activate apoptosis affecting directly both ICER and Bcl2 genes; however, we cannot exclude the involvement of alternative second messengers as for example the modulation of intracellular calcium concentration [54]. The widespread cell apoptosis causes the loss of the microenvironment necessary for the differentiation of hematopoietic stem cells. The consequent failure of erythrocytes production results in an anemia that is probably the principal cause of the death of *PDE2A*^−/−^ embryos.

## 4. Materials and Methods

### 4.1. Mice Husbandry

*PDE2A*^+−-^ mice (B6; 129P2-PDE2A<tm1Dgen>/H; EM:02366) were obtained from EMMA, UK.

Transgenic mice were genotyped using the following primer set:

PDE2A screen F1: 5′-CTGCCTGATGGTGAAGAAAGGCTA-3′,
PDE2A screen F2: 5′-GGGCCAGCTCATTCCTCCCACTCAT-3′,
PDE2A screen R: 5′-TGAGCAGACCCCTTATGGAAGGTG-3′

Briefly, embryo tails were re-suspended in 100 µL of tail buffer (100mM Tris-HCl pH8, 200mM NaCl, 5mM EDTA, 0.2% SDS) containing 0.4 mg/mL proteinase K, heated for 10 min at 95°C and analyzed by Terra PCR Direct Polymerase Mix (Clontech, Mountain View, CA, USA). Breeding cages of C57BL/6 mice were also used to obtained E14.5 embryos for in vitro assays. All our experimental procedures were conforming to the Directive 2010/63/EU of the European Parliament on the protection of animals used for scientific purposes and were conducted with the approval of the Sapienza University’s Animal Use for Research Ethic Committee and by the Italian Ministry of Health with protocol number DGSAF 24675-A (2013).

### 4.2. Histological Procedures and TUNEL Assay

Uteri were removed from pregnant *PDE2A*^+/−^ mice between E11 to E16 and washed with PBS. For successive analysis, individual embryos were processed whole or dissected to remove the liver.

E11.5, E12.5 and E 14.5 embryos or isolated livers were fixed overnight at 4 °C in 4% paraformaldehyde in PBS and embedded in paraffin following standard procedures. Serial sagittal sections were examined after staining with hematoxylin and eosin or underwent to immunofluorescence analysis.

For TUNEL assay, paraffin sections were dewaxed in toluene and antigen retrieval was performed 3 times for 5 min each by microwave heating in 10 mM sodium citrate buffer. After further two washes of 5 min, the sections were incubated in TUNEL reaction mixture (in situ Cell Death Detection Kit, POD, Roche, Basel, Switzerland) for 1 h at 37 °C followed by staining of the nuclei with Hoechst 33342 (Sigma-Aldrich, St. Louis, MO, USA) for 5 min and mounting. Images were acquired by Nikon Eclipse Ti-S microscope.

### 4.3. Micro-CT Imaging and Volume Measurements

Imaging specimen preparation: embryos were washed twice in PBS for 10 min and soaked at room temperature for 1 week in a potassium iodine contrast agent, 0.1N (*v*/*v*) Lugol solution (Sigma-Aldrich), refreshing it twice during the week of exposure (Ermakova et al., 2018). Micro-CT scanning: Computed tomography images were acquired by a high-resolution 3D micro-CT imaging system (Skyscan 1172G Bruker, Kontich—Belgium), using a L7901-20 Microfocus X-ray Source (Hamamatsu). The acquisition of volumes was performed in 1.5 mL micro-tubes, with a camera pixel/size of 7.9 µm, camera binning 2 × 2, tube voltage peak of 39 kV, tube current of 240 µA, exposure time of 450 ms. Reconstructions of tomographic datasets were performed using built-in NRecon Skyscan Software (Version:1.6.6.0; Bruker). The 3D volumes were analyzed using 3D Visualization Software CTvox v. 2.5 (Bruker). Tissues segmentation: Manual image-by-image segmentation aimed at calculation of liver volume was applied, using Bruker micro-CT Analyzer Version 1.13 software. A histological atlas of mouse development [55] was used for guidance to accurately identify, demarcate and segment each liver (*N* = 3) in a specific VOI (Volume of Interest) for automated volume measurements [55,56].

### 4.4. Immunofluorescence

Histological sections were permeabilized in 0.1% Triton *v*/*v*, 3 times for 5 min each and blocked in PBS-3% BSA *w*/*v* for 1 h and incubated with rabbit anti-Ki67 primary antibody (Abcam, Cambridge, UK), rabbit anti-α-Fetoprotein (α-FP) or rabbit α-smooth muscle actin (α-SMA) (all purchased from DAKO-Agilent, Santa Clara, CA, USA). After overnight incubation at 4 °C, sections were washed in PBS and incubated with Alexa Fluor 568 or Alexa Fluor 488 secondary antibody (ThermoFisher scientific, Waltham, MA, USA) for 1 h at room temperature and when specified stained also for TUNEL. Following extensive washes in PBS, the sections were counterstained with DAPI and mounted with PBS-50% *v*/*v* Glycerol. Images were acquired by a Nikon Eclipse Ti-S microscope equipped with a Photometric CoolSNAP EZ turbo 1394 camera.

### 4.5. cAMP Assay

Briefly, E14.5 livers were homogenized and sonicated in cAMP-Glo™ Lysis Buffer (Promega, Madison, WI, USA) containing 0.5 mM IBMX and 100 μM Rolipram (Sigma-Aldrich) following the manufacture’s instruction to prevent cAMP hydrolysis and centrifuged to eliminate cellular debris. Samples were heated for 7–10 min at 70 °C and chilled 5 min in ice. 5 µg from each sample were then mixed with 40 µL of cAMP-Glo Detection Solution containing PKA and incubated for 20 min at room temperature. After the addiction of 80 µL of Kinase-Glo Reagent, the samples were incubated for 10 min and read to the luminometer. Results are referred to a cAMP Standard Curve conducted together with the cAMP-Glo Assay.

### 4.6. Liver Cell Culture and Treatments

Fetal livers were isolated from C57BL/6 E14.5 embryos, mechanically dissociated in phosphate-buffered saline without calcium and magnesium (CMF) (Gibco-ThermoFisher scientific, Waltham, MA, USA) into single cells suspension and centrifuged at 1200 rpm for 5 min at 4 °C. The cellular pellet was re-suspended in DMEM supplemented with 10% FBS and plated in 6 well plates. After 48 h the medium was changed with DMEM 1% FBS with or without EHNA (10 µM, Calbiochem-Sigma-Aldrich, St. Louis, MO, USA) for 24 h. The medium was then changed and supplemented with EHNA (10 µM), or with TNFα (5 ng/mL, Sigma-Aldrich) plus the protein synthesis inhibitor CHX (2.5 µg/mL, Sigma-Aldrich) or with EHNA, TNFα and CHX together. At the end of further 24 h of incubation, the cells were homogenized with lysis buffer and analyzed by western blot or with TRIzol^®^ (Life Technologies, Waltham, MA, USA) reagent for mRNA extraction.

### 4.7. Western Blot Analyses

Western blot analysis was performed on 50–60 µg of proteins from livers homogenized in lysis buffer (25 mM Hepes pH7.4, 0.15% Triton X-100, 150 mM NaCl) containing phosphatase and protease inhibitors. After separation on SDS-PAGE 10% gels proteins were transferred to nitrocellulose membranes (GE Healthcare, Buckinghamshire, UK) that were incubated overnight at 4 °C with rabbit anti-cleaved Caspase (1:200 *v/v* Cell Signaling-9661, USA), or mouse anti-Tubulin (1:2000 *v*/*v*, Sigma-Aldrich-T5168) antibodies and successively with the appropriate horseradish peroxidase-conjugated secondary antibody (Santa Cruz Biotechnology, Dallas, Tex, USA). Chemiluminescent images obtained with an ECL Kit (Santa Cruz Biotechnology, Dallas, Tex, USA) were recorded with the Syngene G-box system (Syngene Bioimaging, India) and immunoblot intensities were quantitatively analyzed using ImageJ Software (NIH, Bethesda, MD, USA).

### 4.8. mRNA Extraction, RT-PCR and qRT-PCR

Total RNA was isolated using TRIzol^®^ reagent (Life Technologies, Waltham, MA, USA) according to manufacturer’s instruction from livers of E12.5 and E14.5 *PDE2A*^+/+^, *PDE2A*^+/−^ and *PDE2A*^−/−^ embryos or from fetal liver cells isolated from E14.5 C57BL/6 embryos and cultured with EHNA as previously described. mRNA was treated with DNAse (Zymo Research, Irvine, CA, USA) and reverse transcribed with random hexamer primers using GoScript (TM) Reverse Transcriptase (Promega, Madison, WI, USA).

After cDNA synthesis step, qRT-PCR reaction was carried out in duplicate for each gene for each liver by using GoTaq qPCR Master Mix (Promega, Madison, WI, USA) using GAPDH for normalization and the reaction carried out on Applied 7500 sequence detection system (Applied Biosystems, Foster city, CA, USA).

Primers pairs used for qRT-PCR are:

cMET fw 5′GATCGTTCAACCGGATCAGAA3′
cMET rev 5′GGAAGAGCCCGGATAATAACAA3′
cEBPα fw 5′AAGAAGTCGGTGGACAAGAACAG3′
cEBPα rw 5′TTGCGTTGTTTGGCTTTATCTC3′
HNF1 fw 5′GCTAGGCTCCAACCTTGTCACG3′
HNF1 rev 5′TTGTGCCGGAAGGCTTCCT3′
HNF4 fw 5′TGGTGTTTAAGGACGTGCTGC3′
HNF4 rev 5′ACGGCTCATCTCCGCTAGCT3′
HNF6 fw 5′AAATAAGCGTCCGTCCAAAGAA3′
HNF6 rev 5′GACGATGAACTGCCTGAGTTG3′
ALBUMIN fw 5′GCTACGGCACAGTGCTTG3′
ALBUMIN rev 5′GTCTTCCACACAAGGCAGTC3′
α-FETOPROTEIN (αFP) fw 5′TCGTATTCCAACAGGAGG3′
α-FETOPROTEIN (αFP) rev 5′AGGCTTTTGCTTCACCAG3′
ICER fw 5′CAAAAGCCCAACATGGCTGT3′
ICER rev 5′GTTACTCTGCTTTATGGCAA3′
BCL2 fw 5′AACCTGTCACAGAGGGGCTA3′
BCL2 rev 5′TGCCGGTTCAGGTACTCAGTC3′
CD31 fw 5′GGTCTTGTCGCAGTATCAGA3′
CD31 rev 5′AGCATTTCGCACACCTGGAT3′
Vimentin fw 5′GTGGAATCCTTGCAGGAAGA3′
Vimentin rev 5′ CAGTGAGGTCAGGCTTGGAA3′
αSMA fw 5′ATGTACCCAGGCATTGCTGA3′
αSMA rev 5′ TTGCTGATCCACATCTGCTG3′
GAPDH fw 5′TGCGACTTCAACAGCAACTC3′
GAPDH rev 5′ATGTAGGCCATGAGGTCCAC3′

### 4.9. Flow Cytometry Analysis

Fetal livers from E14.5 *PDE2A*^+/+^, *PDE2A*^+/−^ and *PDE2A*^−/−^ embryos were collected and separated into single cell suspensions by pipetting in CMF. The cells were re-suspended in FACS buffer (PBS, 2% FBS) and mixed for 1h at 4 °C with the following antibodies: anti-CD45 conjugated in eFluor 450 (BD, Franklin Lakes, N.J., USA); anti-Ter119 FITC (BioLegend, San Diego, CA, USA); anti-F4/80 in phycoerythrin (eBioscience, San Diego, CA, USA); anti-Gr-1 in allophycocyanin (eBioscience), anti-CD41 in Brilliant Violet 421 (Biolegend), anti-CD61 in phycoerythrin (Biolegend, San Diego, CA, USA), anti-CD45R/B220 in FITC (Miltenyi Biotec, Bergisch Gladbach, Germany), anti-CD3 in PE-Cyanine7 (eBioscence), anti-CD11b in PE-Cyanine7 (BD, Franklin Lakes, N.J., USA), anti-CD117 (cKit) in Alexa Fluor 647 (Invitrogen, Waltham, MA, USA), Sca1 FITC (BioLegend). Cells expressing lineage-positive and negative markers were discriminated using a primary antibody cocktail including biotin labeled Ter119, CD45R/B220, CD3e, Gr1 (Ly 6G/Ly 6C) (BioLegend) and a secondary antibody labeled with Brilliant Violet 421 Streptavidin (BioLegend).

For apoptosis, isolated cells were stained with Annexin-V conjugated in FITC (Abcam, Cambridge, UK) and with anti-CD45 conjugated in eFluor 450 (BD, Franklin Lakes, N.J., USA), or anti-CD71 in allophycocyanin (Biolegend) or mouse anti-CD31 (DAKO-Agilent, Santa Clara, CA, USA) reveled with Alexa Fluor 647 secondary antibody (ThermoFisher scientific, Waltham, MA, USA).

Dead cells were stained with 7Amino-Actinomycin D dye (Abcam, Cambridge, UK) and excluded from the analysis.

Analysis was performed with a CyAn cytofluorimeter (DAKO, Santa Clara, CA, USA), Software Summit 4.4 (Beckman coulter).

### 4.10. Cell Cycle Analysis

For cell cycle analysis, 3 × 10^5^ cells for each point were re-suspended in DMEM 50% FBS and fixed in 70% ethanol for 24 h. The cells were then incubated with 50 µg/mL propidium iodide (Sigma-Aldrich) and 50 units/mL Rnase-free DnaseA (Sigma-Aldrich) and analyzed after 3 h (1 × 10^4^ events were acquired) using an Epics XL Cytometer (Beckman Coulter, USA).

### 4.11. Colony-Forming Assay

Individual fetal livers were isolated from E14.5 *PDE2A*^+/+^ and *PDE2A*^−/−^ embryos and mechanically dissociated into single cells suspension in CMF buffer. After centrifugation at 1200 rpm for 5 min at 4 °C, cells were resuspended in IMDM, 2% FBS (Gibco BRL), counted in Trypan Blue (Sigma-Aldrich) and plated in triplicate at 20 × 10^3^ cells/mL, in MethoCult GF 3434 (Stemcell Technology, Vancouver, Canada). Embryonic definitive hematopoietic colonies (BFU-E, CFU-GM, CFU-G CFU-M and CFU-GEMM) were scored after 7 days in a fully humidified incubator at 37 °C with 5% CO_2_. Colonies were counted blindly respect to genotype.

### 4.12. Statistical Analyses

All data are expressed as mean ± SEM and analyzed with one-way ANOVA with Bonferroni correction (Bonferroni Post Hoc Test) or with Student’s *t*-Test. Differences were considered significant if * *P* < 00.5.

## Supplementary Materials

Supplementary materials can be found at https://www.mdpi.com/1422-0067/21/8/2902/s1.

## Abbreviations

PDE2A	Phosphodiesterase 2A
Micro-CT	Micro computational tomography
cAMP	Cyclic adenosine monophosphate
cGMP	Cyclic guanosine monophosphate
E12.5-14.5	Embryonic day
ICER	Inducible cAMP Early Repressor
αFP	Alpha fetoprotein
cMet	Hepatocyte growth factor receptor
cEBP-α	CCAAT/enhancer binding protein alpha
HNFs	Hepatocyte nuclear factors
EHNA	Erythro-9-(2-hydroxy-3-nonly)adenine
CHX	Cycloheximide
TNFα	Tumor necrosis factor-alpha
α-SMA	alpha-Smooth muscle actin
LSK	Lin^−^, Sca-1^+^, c-kit^+^
