# Supplementary material for: PDE2A Is Indispensable for Mouse Liver Development and Hematopoiesis
Source: Int J Mol Sci. 2020 Apr 21;21(8):2902. doi: 10.3390/ijms21082902 (PMC7215450; doi:10.3390/ijms21082902)
Supplement: Supplementary file 1 [file ijms-21-02902-s001.zip › Supplementary Legends.docx]

**Supplementary Video Legends**

**Supplementary Video 1:** Three-Dimensional volume rendering of potassium iodine stained *Pde2A*^+/+^ embryo (E14.5), sectioned in sagittal, coronal and transverse planes.

**Supplementary Video 2:** Three-Dimensional volume rendering of potassium iodine stained *Pde2A^-/-^* embryo (E14.5), sectioned in sagittal, coronal and transverse planes.

**Supplementary S1:** Morphological end molecular defects of the liver increase during development in PDE2A-/- embryos.

**Supplementary S2:** PDE2A inhibition does not affect liver cell differentiation in vitro.

**Supplementary S3:** The percentage of lymphocytes is normal in liver of PDE2A-/- embryos.
