# Supplementary material for: PDE2A Is Indispensable for Mouse Liver Development and Hematopoiesis
Source: Int J Mol Sci. 2020 Apr 21;21(8):2902. doi: 10.3390/ijms21082902 (PMC7215450; doi:10.3390/ijms21082902)
Supplement: Supplementary file 1 [file ijms-21-02902-s001.zip › Supplementary/suppl fig1.pdf]

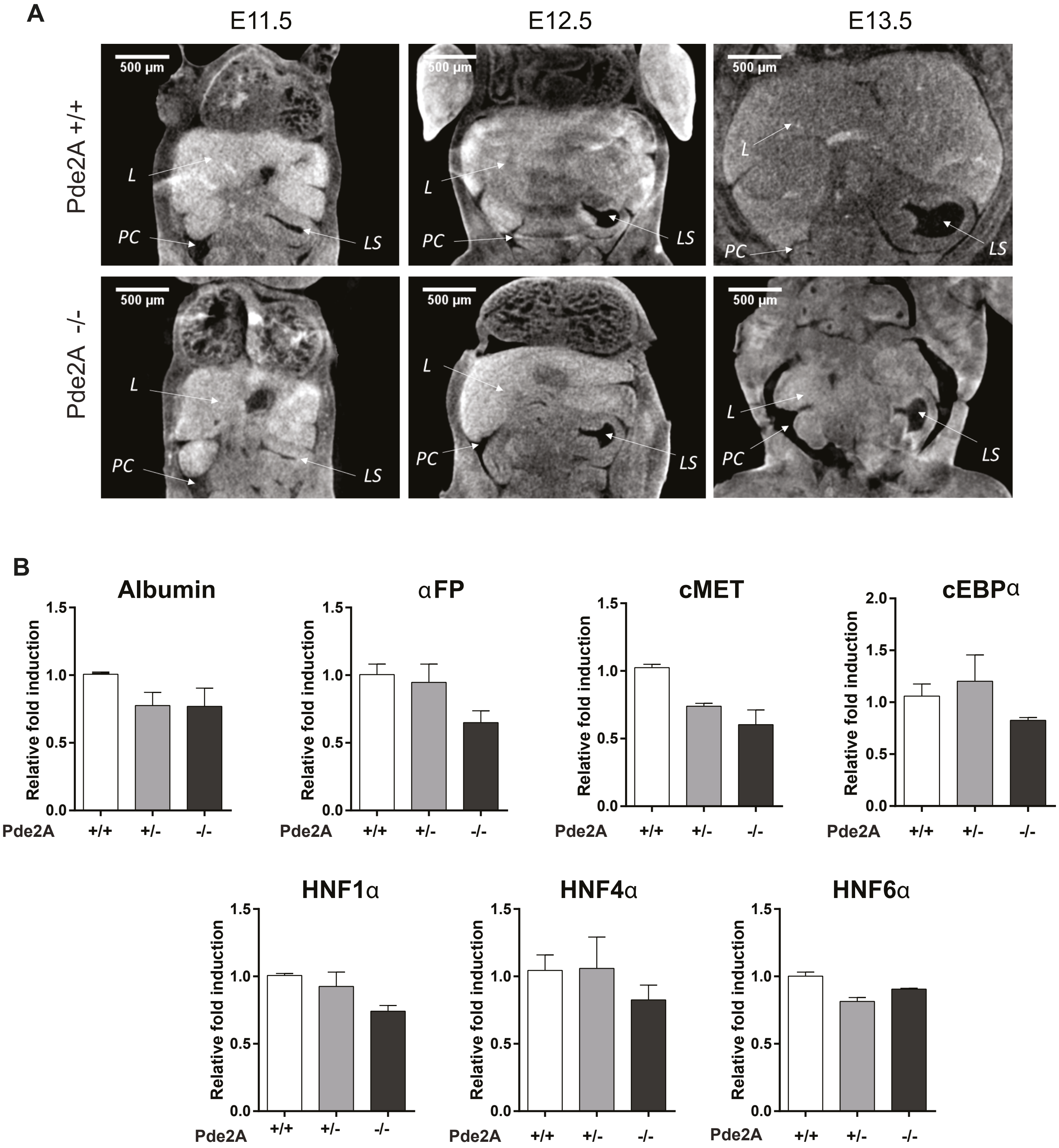

Supplementary Figure 1

Supplementary Figure 1: **(A)** Volume rendering visualization of coronal sections obtained from E11.5, E12.5 and E13.5 *PDE2A*$^{+/+}$and PDE2A$^{-/-}$embryos. Sections were chosen referring to the stomach lumen (*LS*) volume. *L* liver and *PC* peritoneal cavity. Scale bar: 500µm. **(B)** Quantitative RT-PCR in E 12.5 liver embryos shows reduction of expression of liver differentiation markers (albumin; α-fetoprotein) and transcription factors (cMet, cEBPα, HNF1,4) in *Pde2A*$^{-/-}$ embryos. At least N=3 embryos/genotype were analyzed.
