# Supplementary material for: PDE2A Is Indispensable for Mouse Liver Development and Hematopoiesis
Source: Int J Mol Sci. 2020 Apr 21;21(8):2902. doi: 10.3390/ijms21082902 (PMC7215450; doi:10.3390/ijms21082902)
Supplement: Supplementary file 1 [file ijms-21-02902-s001.zip › Supplementary/suppl fig2.pdf]

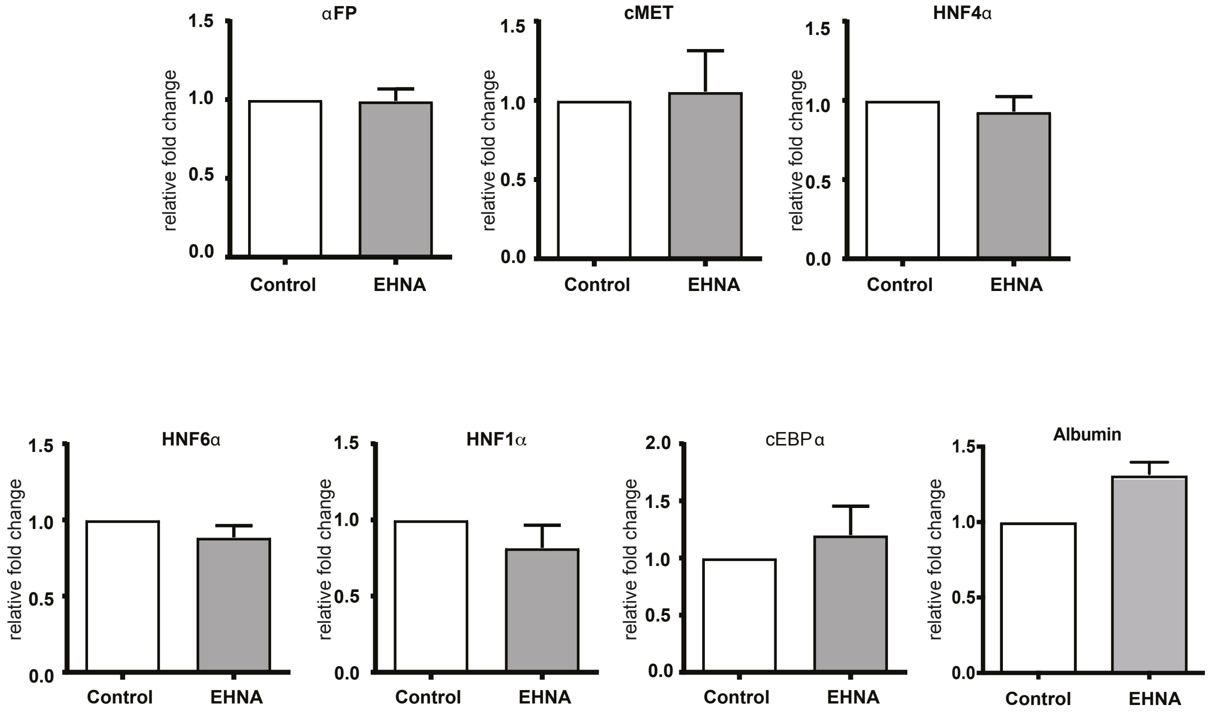

Supplementary Figure 2

Supplementary Figure 2: Quantitative RT-PCR of liver differentiation markers in hepatic cells isolated from E14.5 wild-type embryos after 48h of in vitro treatment with the PDE2A inhibitor EHNA 10µM. N=3.
