# Supplementary material for: PDE2A Is Indispensable for Mouse Liver Development and Hematopoiesis
Source: Int J Mol Sci. 2020 Apr 21;21(8):2902. doi: 10.3390/ijms21082902 (PMC7215450; doi:10.3390/ijms21082902)
Supplement: Supplementary file 1 [file ijms-21-02902-s001.zip › Supplementary/suppl fig3 .pdf]

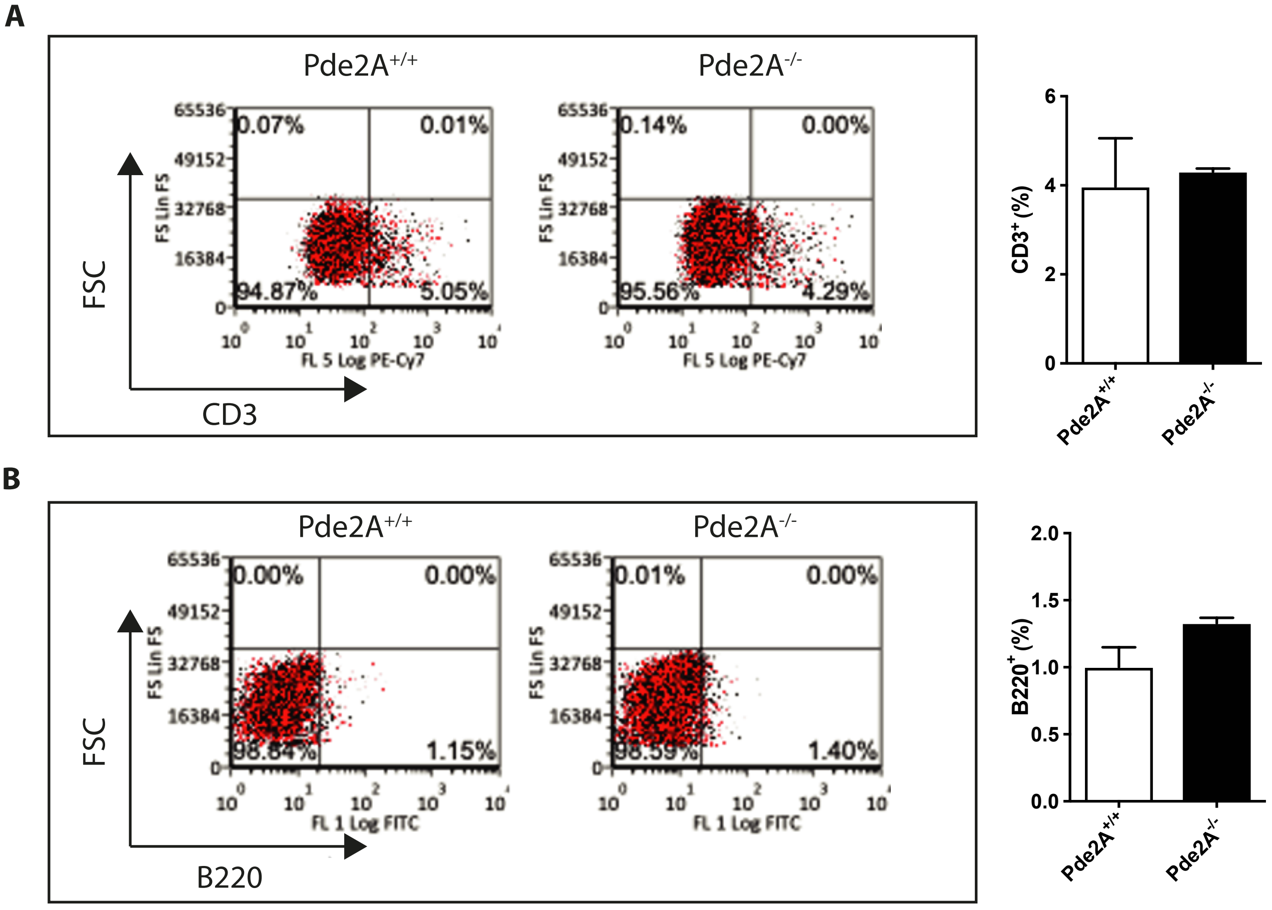

Supplementary Figure 3

Supplementary Figure 3: The percentage of lymphocytes is normal in fetal liver of PDE2A$^{-/-}$ embryos. Panels and histograms show flow cyotmetry analysis of T cells **(A)** and B cells **(B)** isolated from livers of E14.5 embryos and detected with antibodies anti-CD3 and anti-B220, respectively. PDE2A$^{-/-}$ embryos (right panels) compared to PDE2A$^{+/+}$ embryos (left panels) show similar percentage of T and B cells .
